# The clinical importance of the cardiovascular–kidney–metabolic syndrome and related mortality

**DOI:** 10.3389/fcdhc.2025.1719760

**Published:** 2025-12-04

**Authors:** Karolina Hoffmann, Anna Paczkowska, Viviana Maggio, Manfredi Rizzo

**Affiliations:** 1 Department and Clinic of Internal Diseases and Metabolic Disorders, Poznan University of Medical Sciences, Poznań, Poland; 2 Department of Pharmacoeconomics and Social Pharmacy, Poznan University of Medical Sciences, Poznań, Palermo, Poland; 3 Promise Department, School of Medicine, University of Palermo, Palermo, Italy; 4 Ras Al Khaimah Medical and Health Sciences University, Ras Al Khaimah, United Arab Emirates

**Keywords:** cardiovascular-kidney-metabolic (CKM) syndrome, chronic kidney disease, end-stage kidney disease, cardiovascular risk (CV risk), diabetes, obesity

## Introduction

In 1836, Robert Bright first mentioned important structural changes in the heart of patients with advanced kidney disease (1). Nearly 185 years later, the Global Burden of Disease Study 2021 reported the following age-standardized death rates per 100,000: 108.7 (99.8–115.6) for ischemic heart disease, 87.4 (79.5–94.4) for stroke, 19.6 (18.2–20.8) for diabetes, 18.6 (16.9–19.9) for chronic kidney disease (CKD), and 16.3 (14.1–18.0) for hypertensive heart disease (2).

These alarming data inspired the American Heart Association (AHA) to create the term cardiovascular–kidney–metabolic syndrome (CKM), highlighting the multidirectional relationship between cardiovascular disease (CVD), metabolic risk factors, and CKD (3). This novel multisystem disorder is divided into five progressive stages according to the presence of CKM risk factors, ranging from stage 0 (no risk factors) to stage 4 (established CVD) (3).

The key issue is that, due to the increasing prevalence of obesity, diabetes, and atherosclerosis-related diseases, many patients should be classified into advanced CKM stages—yet both they and their physicians are often unaware of the risk (4, 5). Minhas et al., based on U.S. medical data, concluded that the prevalence of advanced CKM ranged from about 10% among individuals aged 45–64 years to over 50% among those over 65 years (6). According to Chen et al., among 110,933 participants from the UK Biobank, nearly 80% were classified into CKM stages 2–4, which are strongly associated with increased risks of both all-cause and CVD-specific mortality (7).

Chen et al. claim that targeted interventions should focus on Caucasian and postmenopausal female patients, who were found to be at higher risk of stages 3 and 4. Conversely, Ostrominski et al. identified older adults, males, non-Caucasian and non-Hispanic ethnicities, and those with socioeconomic disadvantage as being more likely to have clustering CKM conditions compared to other groups (8). Zhu et al. also indicated several unfavorable social determinants of health (SDOH) that increase this risk, including economic status, education level, food security, community and social context, and healthcare system factors (9). Indeed, a comprehensive multi-disciplinary syndemic approach is indicated for the assessment and management of cardiometabolic diseases, in order to take carefully into account the socio-structural environment of any patient (10).

A recently published article also confirmed that CKM health staging is useful for predicting all-cause mortality in Japanese patients with type 2 diabetes (T2DM) (11). All these results indicate that earlier and more comprehensive care—including CKM health staging—is crucial to extending healthy lifespan and improving all-cause mortality in this population. Various confounding factors, such as body composition, fat distribution, and lifestyle, require further investigation.

Recent population-based studies further emphasize the prognostic value of assessing the severity of CKM. In a cohort of over 515,000 adults followed for 16.5 years in Taiwan, Tsai et al. retrospectively demonstrated that the presence and accumulation of CKM components significantly increased the risk of all-cause mortality, cardiovascular mortality, and end-stage CKD (12). Similarly, Li and Wei, using data from the US National Handbook of Epidemiology and Evaluation (NHANES), confirmed a progressive increase in all-cause and cardiovascular mortality in advanced stages of CKM in a prospective cohort study (13). Currently, artificial intelligence (AI) tools are becoming a potential medical aid in early identification of CKM risk and personalized treatment by integrating metabolic, renal, and cardiovascular data. However, this approach requires rigorous clinical validation and ethical implementation to complement clinicians’ judgment and improve preventive strategies in the care of patients with CKM.

## Current therapy management

Current non-pharmacological recommendations for CKM focus on lifestyle modification: proper nutrition, regular physical activity, sleep hygiene, avoiding smoking and excessive alcohol, and caring for mental health. The multidisciplinary team should educate patients in all aspects of therapy and encourage them that any effort they make will be worthwhile. The team should ideally include physicians, physician assistants, nurses, dietitians, exercise therapists, mental health specialists, and social workers.

In general, interventions for CKM stages 0 to 3 aim to prevent future CV complications by managing overweight and obesity according to current guidelines (14). An optimal, tailored diet and exercise program are sufficient in stage 1, whereas pharmacological treatment of metabolic syndrome components should be initiated in stage 2. In stage 3 CKM (subclinical CVD), therapy should comprehensively address all CKM components. In stage 4, all components of advanced CKM should be treated according to the relevant guidelines (15).

Fortunately, people diagnosed with advanced CKM stages can benefit from a broad spectrum of therapeutic options to reduce their risk. Lipid-lowering therapy should be individualized to achieve risk-based low-density lipoprotein cholesterol (LDL-C) targets (14). Physicians should also aim for blood pressure <130/80 mmHg in hypertensive patients (15). The choice of antithrombotic drug depends on the presence of risk factors and diagnosed CV conditions (15).

Glucose-lowering therapy should be tailored to each patient’s glycemic goals. Sodium–glucose cotransporter-2 inhibitors (SGLT2i) or glucagon-like peptide-1 receptor agonists (GLP-1 RA) are indicated independently of glycemic control in patients with T2D who have or are at risk of ASCVD, CKD, and/or heart failure (HF). Evidence from randomized controlled trials (RCTs) confirms that SGLT2i and GLP-1 RA reduce cardiorenal risk and related mortality independently of their glucose-lowering effect (16, 17). It has been further shown that GLP-1 RA are able to reduce the formation and progression of atherosclerosis due to a direct anti-atherogenic effect in lowering small dense low-density lipoproteins (LDL), particles that are closely associated to cardiovascular events (18). By contrast, antidiabetic agents without cardiovascular benefit have shown no effect on atherogenic lipids such as small, dense LDL (19).

Additionally, the non-steroidal MRA finerenone demonstrated CV and kidney benefits in diabetic patients in two recent trials: FIDELIO-DKD and FIGARO-DKD (20). Dual GLP-1/GIP receptor agonists, such as tirzepatide, can increase the odds of achieving normoglycemia by >16-fold, corresponding to glycated hemoglobin levels <5.7% (21). There is also growing evidence for tirzepatide’s benefits in metabolic dysfunction–associated steatotic liver disease (MASLD), obstructive sleep apnoea–hypopnoea syndrome (OSAHS), and HF (21, 22).

## Innovative pharmacological treatments for CKM

The development of new drugs aims to improve treatment outcomes for patients with CKM and extend lifespan. Among novel therapies are triple hormone receptor agonists (GLP-1/GIP/glucagon), lysophosphatidic acid (LPA) receptor antagonists, and second-generation selective MRAs combined with SGLT2i (23, 24).

Triple hormone receptor agonists, such as retatrutide, are expected to enhance energy expenditure and improve lipid metabolism, promising greater reductions in liver fat and earlier achievement of glucose balance (23, 24). Preclinical trials with LY3437943 have shown similar metabolic benefits, but its long-term safety and efficacy require confirmation. LPA receptor antagonists target the LPA1 receptor, involved in CKD and HF pathogenesis. Ongoing studies with BMS-986020 suggest significant antifibrotic effects (23, 24).

Two second-generation non-steroidal MRAs—balcinrenone and esaxerenone—are being evaluated for CKD and HF. The phase 3 trial ESAX-DN in hypertensive patients marked the end of esaxerenone’s development, while the MIRO-CKD (NCT06350123) and BALANCED HF (NCT06307652) trials are assessing balcinrenone combined with dapagliflozin. Combination therapies potentially effective in CKM, targeting several disease pathways, are also under investigation in the EASi-KIDNEY trial (vicadrostat + empagliflozin) and the ZENITH-CKD trial (zibotentan + dapagliflozin versus dapagliflozin alone) (23, 24).

## Conclusion: the importance of prevention in CKM

In conclusion, there is a global need for effective public health interventions to diagnose CKM early and prevent its long-term consequences. Prevention remains crucial in CKM management. Early detection and interventions targeting inflammation, obesity, and insulin resistance are key to optimizing treatment outcomes and reducing mortality associated with CKM. Neither novel therapies nor technological innovations, such as digital health tools, alone can improve both the quality and quantity of life for our patients. As clinicians we have the responsibility to check the CKM stage of our patients. We may not fully stop CKM’s spread, but early action can change its trajectory.
